# Highly Sensitive NO_2_ Gas Sensors Based on ZnO-Coated Black Silicon Nanostructures

**DOI:** 10.3390/nano16070427

**Published:** 2026-03-31

**Authors:** Gagik Ayvazyan, Laura Lakhoyan, Alina Semchenko, Vazgen Melikyan

**Affiliations:** 1 Optoelectronics Laboratory, National Polytechnic University of Armenia, 105, Teryan Street, Yerevan 0009, Armenia; llakhoyan@mail.ru (L.L.); vazgen.meliqyan@synopsys.com (V.M.); 2 Department of Physics and Information Technologies, Skorina Gomel State University, 104, Sovetskaya, 246019 Gomel, Belarus; alina@gsu.by

**Keywords:** zinc oxide, black silicon, gas sensor, nitrogen dioxide, sol–gel deposition

## Abstract

We report highly sensitive NO_2_ gas sensors based on ZnO thin films prepared via a sol–gel method and deposited onto nanostructured black silicon (b-Si). The b-Si layers, fabricated using maskless reactive ion etching, consist of densely packed silicon nanoneedles with an average height of ~810 nm, a base diameter of ~160 nm, and a characteristic periodicity of ~190 nm. Owing to this highly developed surface morphology, the effective surface area of the b-Si layer is estimated to be approximately one order of magnitude higher than that of planar silicon, thereby enhancing gas adsorption and charge-transfer processes in the ZnO film. ZnO/b-Si/Si sensors exhibit a response of 448% at 25 ppm NO_2_ at an optimal operating temperature of 200 °C, which is approximately 1.5 times higher than that of planar ZnO/Si sensors at the same concentration and temperature. Notably, a comparable response (~300%) is achieved at a reduced temperature of 140 °C, indicating the potential for low-power operation. The sensing mechanism is governed primarily by the ZnO layer, while b-Si serves as a morphological scaffold, increasing the effective surface area. These results demonstrate that ZnO-coated b-Si nanostructures represent a promising platform for high-performance NO_2_ sensing and offer strong potential for integration with silicon-based microelectronic technologies.

## 1. Introduction

Among various toxic and environmentally hazardous gases, nitrogen dioxide (NO_2_) is of particular concern due to its high chemical reactivity, toxicity even at low concentrations, and widespread presence in vehicle exhaust emissions, power generation systems, and chemical industry processes. NO_2_ contributes to the formation of photochemical smog and acid rain and adversely affects human respiratory health as well as biological processes in vegetation. Consequently, the development of highly sensitive, selective, and long-term stable sensors for NO_2_ detection that are capable of operating at reduced temperatures remains a critical scientific and technological challenge of significant environmental and societal importance.

Owing to their compact size, low manufacturing cost, and operational simplicity, transition metal dichalcogenide (TMD) [1] and metal-oxide semiconductor (MOS) [2,3] sensors represent efficient platforms for detecting gas molecules, including NO_2_. Zinc oxide (ZnO) is one of the most widely studied MOS materials for gas sensing due to its high electron mobility, chemical and thermal stability, non-toxicity, and compatibility with micro- and nanoelectronic fabrication technologies [4,5,6,7]. Its wide bandgap also makes it attractive for optoelectronic applications. However, in resistive gas sensors, the key factor is the combination of semiconductor properties with the controlled formation of nanostructured films, including nanowires, nanorods, and nanosheets, as well as the surface modification with various nanoparticles [8,9,10,11]. The near-surface conductivity of ZnO is highly sensitive to adsorption of charged species, such as ionized oxygen or NO_2_ molecules, and its high density of surface active sites (e.g., oxygen vacancies) underpins its leading performance among sensing materials.

Various fabrication techniques have been developed for ZnO nanostructures, including electrospray deposition [12], atomic layer deposition [13], magnetron sputtering [14], chemical vapor deposition [15], thermal evaporation [16], and the sol–gel method [17,18]. The sol–gel approach is particularly attractive due to its simplicity, relatively low processing temperature, and the ability to form uniform films on substrates with complex morphologies while maintaining precise control over film thickness and nanostructure.

A common approach to enhancing the sensitivity of MOS gas sensors is to use structured substrates that increase the specific surface area and modify the electronic properties of the active layer. In this context, porous silicon has been widely studied, which forms a microstructured network of branched pores through electrochemical or chemical etching. Its large surface area improves sensor response and can reduce operating temperatures, sometimes even to room temperature [19,20]. Several studies have shown that thin-film ZnO sensors fabricated on porous silicon substrates exhibit higher sensitivity toward NO_2_ compared with their counterparts on planar silicon substrates [21,22,23,24]. A similar strategy has also been applied to other thin-film gas-sensitive materials such as V_2_O_5,_ WO_3_, MoS_2_, CuO, and SnO_2_ for the detection of CO_2_, NO_2_, SO_2_, H_2_S, and volatile organic compounds [25,26,27,28,29]. However, the presence of micron-scale pores and complex morphology limits the uniform penetration of the sensing material and diffusion of gas molecules to active sites. Additionally, the wet fabrication processes for porous silicon are often incompatible with standard microelectronic technologies, complicating integration with electronic circuits.

Nanostructured black silicon (b-Si) offers a promising alternative. It consists of densely packed arrays of high-aspect-ratio nanoneedles, providing an extremely high surface area and fully open pathways for gas diffusion. When fabricated by dry techniques such as reactive ion etching (RIE), b-Si exhibits excellent compatibility with modern microelectronic fabrication [30]. Most studies of b-Si focus on optoelectronic applications, including solar cells, photodiodes, and photodetectors [31,32,33,34,35,36,37]. Recent studies have explored b-Si for optical, electrical, and hybrid gas sensors, often enhanced by thermal or optical treatments or by hyperdoping with elements such as nitrogen, sulfur, or scandium [38,39,40,41]. The use of pristine b-Si in resistive gas sensors was previously demonstrated by our group [42]. However, despite its potential, the application of pristine or modified b-Si in resistive MOS gas sensors remains largely unexplored.

In this work, we experimentally demonstrate a highly sensitive NO_2_ sensor based on ZnO thin films deposited via the sol–gel method onto a nanostructured b-Si layer. We establish correlations between the b-Si morphology, the structural and phase characteristics of ZnO films, and the resulting gas-sensing performance, and identify the key factors responsible for the synergistic enhancement of sensor sensitivity and the reduction in operating temperature.

## 2. Experimental Section

### 2.1. Sensor Fabrication

**Figure 1:**
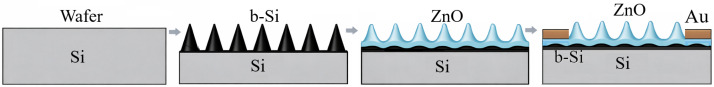
Schematic of the fabrication process of ZnO/b-Si/Si sensors.

Figure 1 shows a step-by-step schematic of the fabrication process of gas sensors with front contacts based on ZnO films deposited on a nanostructured b-Si layer by the sol–gel method. Hereafter, these structures are referred to as ZnO/b-Si/Si. This notation describes the three-layer structure consisting of the upper ZnO sensing layer, the intermediate nanostructured b-Si layer, and the silicon wafer.

Monocrystalline silicon wafers with p-type conductivity, (100) orientation, and a resistivity of 1.0–3.0 Ω·cm were used as starting substrates. Before processing, the wafers were immersed in an HF solution for 10 s to remove the native oxide film, followed by rinsing in ethanol and drying in a nitrogen flow. The b-Si layer was formed on the front side of the substrates by a maskless RIE method in an SF_6_/O_2_ gas mixture. The process was carried out at a pressure of 55 mTorr for 13 min with gas flow rates of 75 sccm (SF_6_) and 35 sccm (O_2_). This regime was previously optimized for the fabrication of resistive gas sensors based on pristine b-Si [42]. After etching, the sample surfaces were rinsed with distilled water and dried in a nitrogen flow.

The fabrication of ZnO films was carried out using a sol–gel method and consisted of two consecutive stages. In the first stage, a ZnO seed layer was deposited on the substrates. The sol was prepared by dissolving 1.5 g of zinc acetate Zn(CH_3_COO)_2_ in 8 mL of isopropyl alcohol. The resulting sol was aged for 2–3 days at room temperature. Isopropyl alcohol served as a dispersion medium, ensuring solution stability and appropriate viscosity for spin coating. The sol was deposited onto the b-Si layer by spin coating at a rotation speed of 2000 rpm. After deposition, the samples were subjected to preliminary annealing at 250 °C for 5 min in a muffle furnace. Final annealing was performed at 350 °C for 60 min, resulting in the formation of a uniform ZnO seed layer with a thickness of approximately 30 nm.

ZnO nanostructures were subsequently synthesized by chemical deposition from an aqueous solution. A 0.2 M aqueous solution of zinc acetate Zn(CH_3_COO)_2_ was added dropwise under continuous stirring into a reaction vessel containing 200 mL of distilled water. After achieving a homogeneous solution, 1 mL of ethylenediamine was added, increasing the pH to approximately 8.5 and initiating the precipitation process. The substrates with the pre-formed ZnO seed layer were immersed in the solution immediately before the addition of ethylenediamine. The reaction vessel was hermetically sealed and maintained in a drying oven at 90 °C for 2 h. After the process, the samples were rinsed with distilled water and dried in air. The ZnO nanorods formed on the seed layer had an average length of 65 nm. Thus, the total thickness of the ZnO sensing film was approximately 95 nm. This thickness ensures conformal coverage of the b-Si nanoneedles without filling the inter-needle gaps, thereby preserving the high effective surface area of the structure.

To form interdigitated contacts, a 100 nm-thick gold (Au) film was deposited onto the ZnO surface through a shadow mask by thermal vacuum evaporation. The substrates were then diced into individual sensor elements with dimensions of 2.0 × 2.0 cm^2^. Electrical connections were formed by attaching copper wires with a diameter of 0.3 mm to the contact pads using indium.

**Figure 2:**
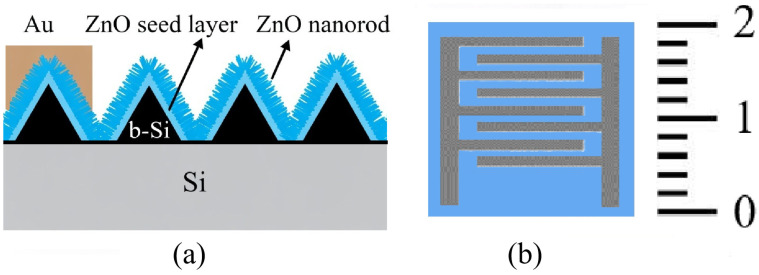
Schematic representation: (**a**) Structure; (**b**) Front contacts. Photograph of the fabricated device.

Figure 2 shows schematic representations of the sensor structure and front contacts. of the fabricated device. For comparison, reference sensors were also fabricated on planar silicon wafers without the b-Si layer. These structures are referred to as ZnO/Si.

### 2.2. Characterization and Gas Sensing Measurements

The morphology of the samples, including surface and cross-sectional features, was examined by scanning electron microscopy (SEM) using a Hitachi S-4800 microscope (Hitachi, Tokyo, Japan). The elemental composition was analyzed by energy-dispersive X-ray spectroscopy (EDS) using a Bruker QUANTAX200 spectrometer (Mannheim, Germany) at an accelerating voltage of 10 kV. Phase and structural analysis were performed by X-ray diffraction (XRD) using a Malvern Panalytical diffractometer (Almelo, The Netherlands) with CuKα radiation (λ = 1.5406 Å) in 2θ scanning mode.

**Figure 3:**
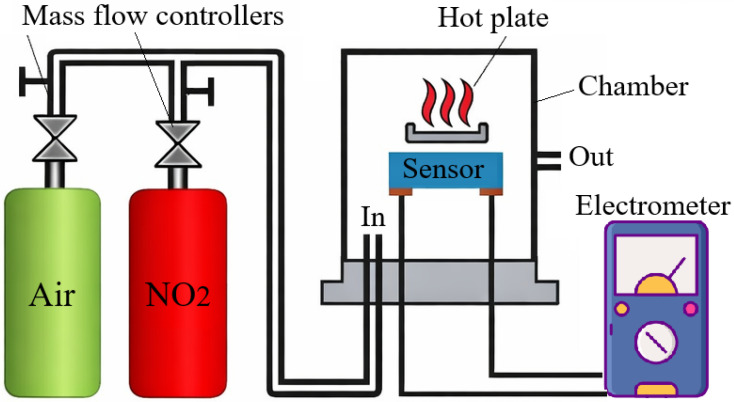
Schematic illustration of the experimental setup for gas sensing measurements.

The gas-sensing properties of the fabricated sensors were investigated at various concentrations of NO_2_ in the operating temperature range of 125–250 °C using a laboratory-built experimental setup as schematically shown in Figure 3. The sensors were placed on a PID-controlled hot plate inside a sealed chamber with a volume of 3 L equipped with gas inlet and outlet ports. Dry compressed air was used as the carrier, diluting, and purging gas. Flow rates of air and NO_2_ were independently controlled by mass flow controllers. Current–voltage (I-V) characteristics of the sensors were recorded in real time using an electrometer E7-25 (MINIPI, Minsk, Belarus).

The sensor response $(S_{R}$) was defined as the relative change in electrical resistance, calculated from the I–V characteristics as follows:

$$S_{R}=\frac{R_{gas}-R_{air}}{R_{air}}\times 100\%,$$

where $R_{gas}$ is the electrical resistance of the sensor at a given NO_2_ concentration and $R_{air}$ is the resistance measured in dry air. The response time ($t_{\mathrm{res}}$) and recovery time ($t_{\mathrm{rec}}$) were defined as the time required for the sensor resistance to reach 90% of the total change after the introduction and removal of the target gas, respectively.

## 3. Results and Discussion

### 3.1. Structural and Morphological Properties

**Figure 4:**
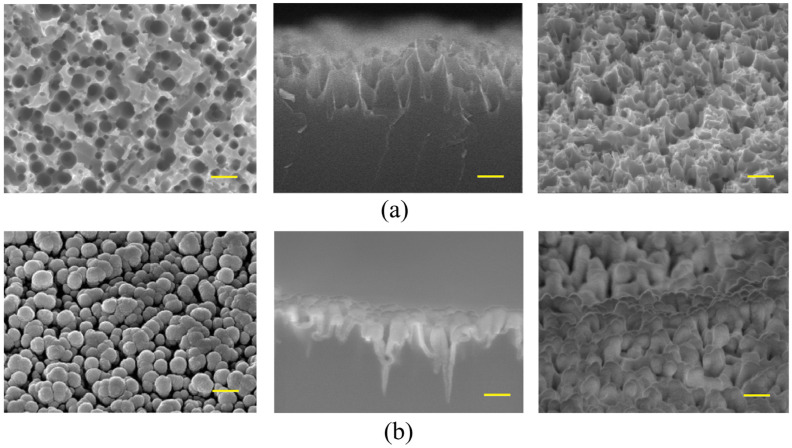
SEM images (top view, cross-sectional view, and a tilted view at 70°) of the b-Si layers: (**a**) Before and (**b**) after ZnO deposition. All scale bars are 500 nm.

Figure 4 shows representative SEM images (top view, cross-sectional, and 70° tilted views) of the b-Si layers before and after the deposition of ZnO films. The surface morphology of the b-Si layer exhibits a highly developed nanostructured architecture formed by densely packed silicon nanoneedles. The nanoneedles are vertically oriented and uniformly distributed across the surface, forming a quasi-periodic array. Statistical analysis of the SEM images indicates that the nanoneedles have an average height of ~810 nm, a base diameter of ~160 nm, and a characteristic periodicity of ~190 nm. When modeled as a hexagonal array of right circular cones with these parameters, the b-Si layer provides an effective surface area approximately one order of magnitude larger than that of a planar silicon wafer.

The ZnO film conformally coats the silicon nanoneedles, preserving the underlying b-Si morphology. This coating increases the ZnO/b-Si interfacial area and increases the active surface area for gas adsorption, promoting efficient charge transfer and enhancing the gas sensing performance of ZnO/b-Si/Si sensors.

**Figure 5:**
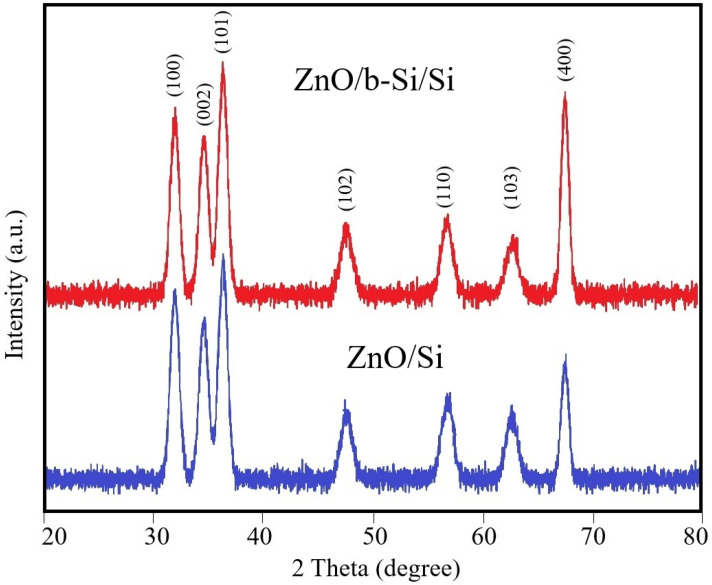
XRD patterns of ZnO films deposited on planar silicon (ZnO/Si) and b-Si layers (ZnO/b-Si/Si).

Figure 5 presents the X-ray diffraction (XRD) patterns of ZnO films deposited on planar silicon surfaces and on b-Si layers. In both cases, pronounced diffraction peaks corresponding to the (100), (002), (101), (102), (110), and (103) crystallographic planes of hexagonal wurtzite ZnO (JCPDS Card No. 36-1451) are observed in the 2θ range of 20–80°, confirming the phase purity of the deposited films. The peak at 2θ ≈ 68.1° corresponds to the silicon wafer. The higher intensity of this peak in ZnO/b-Si/Si compared to ZnO/Si reflects the preservation of crystalline silicon in the nanoneedles, contributing to the diffraction signal. In contrast, the ZnO peak intensities are slightly lower for the thinner films (~95 nm) compared with thicker films (~530 nm) reported previously [43], attributed to the smaller volume fraction of crystalline ZnO.

The average crystallite size of ZnO was estimated from the (101) diffraction peak using the Scherrer equation:

$$D=\frac{k\lambda }{\beta cos\theta },$$

where D is the average crystallite size, k is the Scherrer constant, β is the full width at half maximum (FWHM) of the diffraction peak, and λ is the wavelength of the X-ray radiation.

The calculations show that the average crystallite size is approximately 22 nm for ZnO films deposited on planar silicon substrates and about 27 nm for films deposited on the b-Si layer. The increase is likely due to the high lyophilicity of the b-Si surface [44], which facilitates improved penetration of the zinc precursor solution and promotes grain growth during sol–gel synthesis. The obtained crystallite sizes are smaller than those reported for thicker ZnO films (~530 nm) [43], which can be attributed to differences in crystallization kinetics associated with the reduced film thickness, limiting the time and space available for lateral grain growth.

Table 1 summarizes the quantitative elemental composition of the experimental samples determined by EDS analysis (the uncertainty for the main components does not exceed ±0.5 at.%). The analysis shows that the Zn/O ratio is close to the stoichiometric composition of ZnO; however, the absolute concentrations represent a combined signal from both the film and the substrate due to the relatively large penetration depth of the generated characteristic X-ray radiation.

For the ZnO/b-Si/Si structure, an increase in the relative concentrations of Zn and O is observed compared to the ZnO/Si structure, which is consistent with a higher amount of deposited material on the highly developed surface of the substrate. Dopants in the substrate (phosphorus) and potential adsorbed carbon or nitrogen were below the EDS detection limit. Elemental mapping confirms a uniform Zn distribution, indicating high homogeneity of the films. Compared with thicker films (~530 nm) [43], the thinner films (~95 nm) exhibit a higher relative contribution from the silicon substrate signal due to reduced X-ray attenuation.

### 3.2. Gas Sensing Properties

**Figure 6:**
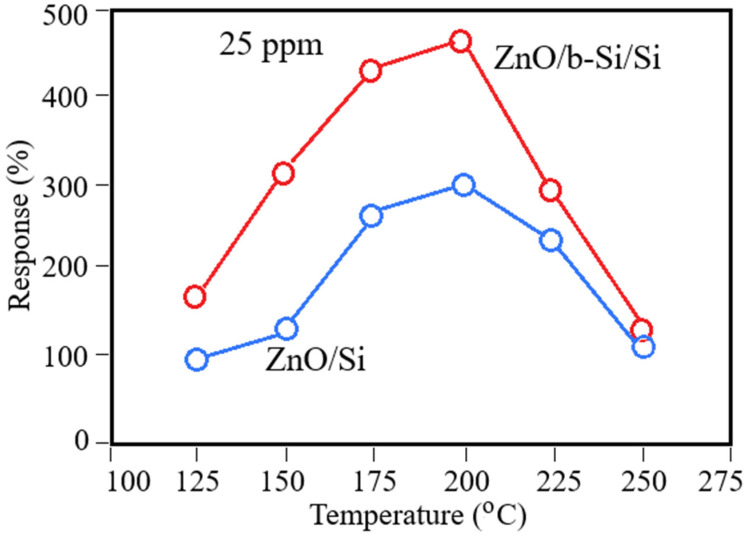
Temperature dependence of the sensor response at 25 ppm NO_2_.

Optimization of the operating temperature is a key step in the development of resistive gas sensors based on MOSs, since gas adsorption, surface redox reactions, and desorption are strongly temperature-dependent. Figure 6 shows the temperature dependence of the sensor response at a fixed NO_2_ concentration of 25 ppm. Both ZnO/Si and ZnO/b-Si/Si structures exhibit a characteristic bell-shaped response typical of ZnO-based sensors: the response increases with temperature, reaches a maximum at the optimal operating temperature, and then gradually decreases [4,5,6]. Notably, the maximum response for both structures occurs at ~200 °C, suggesting that the b-Si layer primarily increases the number of active adsorption sites without significantly altering the activation energy of surface reactions.

The maximum response observed at approximately 200 °C results from an optimal balance between the temperature-dependent adsorption of NO_2_ molecules, the kinetics of surface reactions, and charge transport in the ZnO sensing layer. At lower temperatures, insufficient thermal energy limits adsorption kinetics and surface reactions. In contrast, at higher temperatures (225–250 °C), the enhanced desorption of NO_2_ molecules reduces the concentration of adsorbed species on the ZnO surface, resulting in a decrease in sensor response.

As seen in Figure 6, the incorporation of the b-Si layer significantly enhances the sensor response across the entire temperature range. At 200 °C, ZnO/b-Si/Si sensors exhibit a response of 446%, approximately 1.5 times higher than that of planar ZnO/Si sensors. A comparable response (~302%) for planar ZnO/Si is achieved in ZnO/b-Si/Si sensors already at 140 °C, demonstrating that b-Si enables a substantial reduction (~60 °C) in operating temperature while maintaining high sensitivity. This reduction in operating temperature is beneficial for lowering the power consumption of the sensor, as the power required for microhotplate-based devices scales approximately linearly with the temperature difference above ambient. Achieving comparable sensitivity at a lower temperature, therefore, offers a pathway toward energy-efficient operation, which is particularly important for portable and low-power gas sensing applications.

The higher sensitivity of the ZnO/b-Si/Si sensors across the entire investigated temperature range is primarily explained by the significantly larger effective surface area of the nanostructured b-Si layer. This, in turn, leads to the formation of a ZnO film with a greater real gas-sensitive surface area due to its coating along the sidewalls of the nanoneedles. Additionally, a contributing factor may be the modification of the defect structure of the ZnO film at the interface with the b-Si. Collectively, these factors provide a greater number of active sites for NO_2_ adsorption and subsequent charge transfer.

All subsequent gas sensing measurements were performed at the optimal operating temperature in order to ensure reliable comparison of the sensor characteristics and to more accurately evaluate the contribution of the nanostructured b-Si layer to the enhancement of the NO_2_ sensing performance.

**Figure 7:**
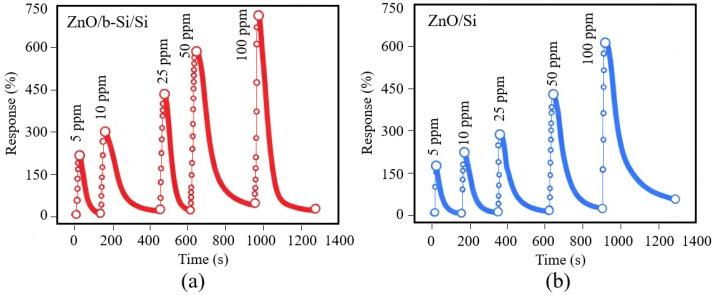
The dynamic sensing response: (**a**) ZnO/b-Si/Si; (**b**) ZnO/Si.

Dynamic sensing measurements were performed in the NO_2_ concentration range of 5–100 ppm. Time-dependent resistance changes were recorded under cyclic NO_2_ exposure followed by dry air purging. Figure 7 shows the reproducible and reversible sensor response across all concentrations.

With increasing NO_2_ concentration, a monotonic increase in sensor response is observed, indicating that adsorption-induced surface processes dominate the conductivity modulation in the ZnO sensing layer. The response of ZnO/b-Si/Si sensors at NO_2_ concentrations of 5, 10, 25, 50, and 100 ppm is 173%, 302%, 448%, 591%, and 728%, respectively. In comparison, ZnO/Si sensors fabricated on planar substrates exhibit response values of 167%, 229%, 298%, 451%, and 613% at the same NO_2_ concentrations. At low NO_2_ concentration (5 ppm), the difference between the responses of the two sensor types is relatively small, which may be attributed to incomplete utilization of the additional adsorption sites introduced by the nanostructured b-Si surface. As the gas concentration increases, the contribution of the enlarged surface area becomes more pronounced, resulting in a progressively larger difference in sensor response.

**Figure 8:**
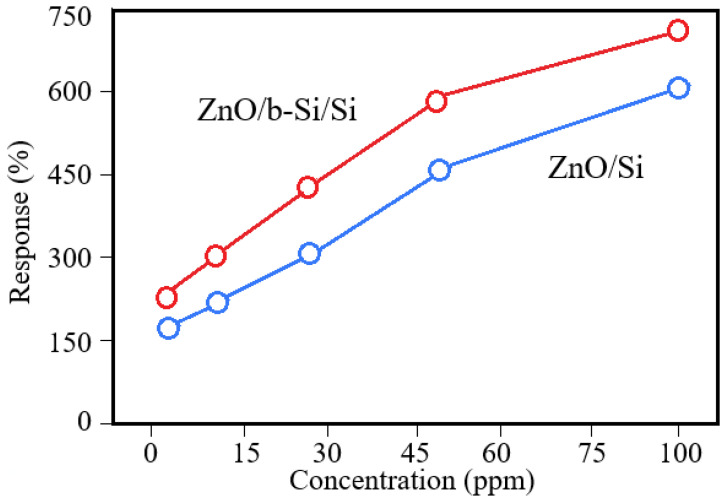
Sensor response vs. NO_2_ concentration.

The dependence of the sensor response on NO_2_ concentration (Figure 8) in the range of 5–50 ppm is well described by a linear function (R^2^ > 0.98), which is an important factor for the practical application of these sensors in early toxic gas detection systems, allowing for quantitative concentration assessment. Based on this linearity, it can be estimated that for the ZnO/b-Si/Si sensors at 0.5 ppm, the response would be in the range of 170–180%; however, a rigorous determination of the limit of detection would require additional measurements at sub-ppm concentrations. The observed linearity indicates the absence of saturation of active adsorption sites on the ZnO surface within this concentration range. At higher concentrations (100 ppm), a tendency toward a slower increase in the sensor response is observed, which may be associated with the onset of saturation of the available adsorption sites.

**Figure 9:**
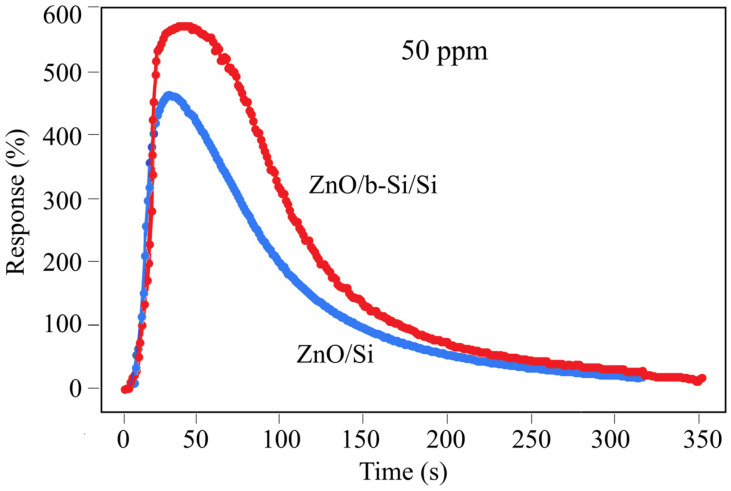
Dynamic temporal response of ZnO/Si and ZnO/b-Si/Si sensors at 50 ppm NO_2_.

Figure 9 shows the temporal response of ZnO/Si and ZnO/b-Si/Si sensors at a concentration of 50 ppm NO_2_. For the ZnO/Si sensors, the response and recovery times are 19 and 218 s, respectively, whereas for the ZnO/b-Si/Si sensors, these values increase to 34 and 296 s. Thus, the incorporation of a nanostructured b-Si layer increases the response and recovery times of the sensors. The observed increase in response and recovery times for the ZnO/b-Si/Si sensors is likely due to a combination of two factors. First, it is associated with diffusion limitations, as the ZnO nanorods on the sidewalls of the nanoneedles are not oriented vertically with respect to the gas flow. In this case, gas molecules require more time to penetrate into and out of the deeper regions of the ZnO film. Second, the high density of defects and active sites on the extended ZnO surface may lead to the formation of stronger bonds with NO_2_ molecules, which increases sensitivity but simultaneously slows down the desorption process, resulting in longer recovery times. Such a trade-off between high sensitivity and fast recovery is a characteristic feature of sensors with highly developed surfaces.

**Figure 10:**
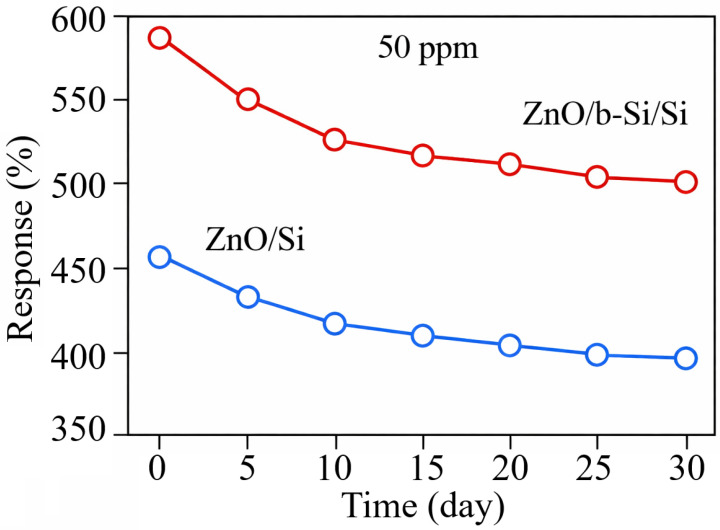
Long-term stability of ZnO/Si and ZnO/b-Si/Si sensors at 50 ppm NO_2_.

From the perspective of practical sensor applications, long-term stability under natural aging of the sensitive material is another important factor. Figure 10 shows the effect of aging on the gas-sensing properties of ZnO/Si and ZnO/b-Si/Si sensors upon exposure to 50 ppm NO_2_ over a period of 30 days, measured at 5-day intervals. The results show that during the first 10 days of storage, the response of both sensors decreases, after which the response changes slowly. Importantly, both sensors exhibited a stability of 80–85%, indicating that the presence of the b-Si layer has virtually no adverse effect on sensor stability.

The gas sensing mechanism of the investigated sensor structures can be explained by surface adsorption processes and charge transfer occurring in the ZnO sensing layer [45,46]. Zinc oxide is a typical n-type semiconductor whose gas sensing properties are mainly governed by the presence of oxygen vacancies and surface defect states that facilitate the adsorption of gas molecules and subsequent charge exchange. When the sensors are exposed to air, oxygen molecules are adsorbed on the surface of the ZnO film and capture electrons (*e*^−^) from the conduction band, forming ionized oxygen species ($O_{2}^{\;-}$, $O^{-}$) according to the following reactions:

$$O_{2}(gas)\to O_{2}(ads),$$

$$O_{2}\left(ads\right)+e^{-}\to O_{2}^{-}\left(ads\right),$$

$$O_{2}^{-}\left(ads\right)+e^{-}\to 2O^{-}\left(ads\right).$$

As a result of this process, an electron depletion layer (EDL) is formed near the ZnO surface, accompanied by band bending and an increase in the potential barrier for charge carrier transport. This leads to a decrease in the concentration of free electrons in the conduction channel of ZnO and, consequently, to an increase in the electrical resistance of the sensing layer.

Upon exposure to NO_2_, which is a strong oxidizing gas, additional electron trapping occurs at the ZnO surface. NO_2_ molecules can either adsorb directly on the ZnO surface or interact with previously adsorbed oxygen species:

$${NO}_{2}(gas)+e^{-}\to {NO}_{2}^{\;-}(ads),$$

$${NO}_{2}\left(gas\right)+{O^{-}\left(ads\right)+e}^{-}\to {NO}_{3}^{-}\left(ads\right).$$

These reactions lead to further extraction of electrons from the conduction band of ZnO, resulting in an expansion of the EDL. Consequently, the height of the potential barrier increases and the effective width of the conductive channel decreases, which manifests as a significant increase in the electrical resistance of the sensor.

**Figure 11:**
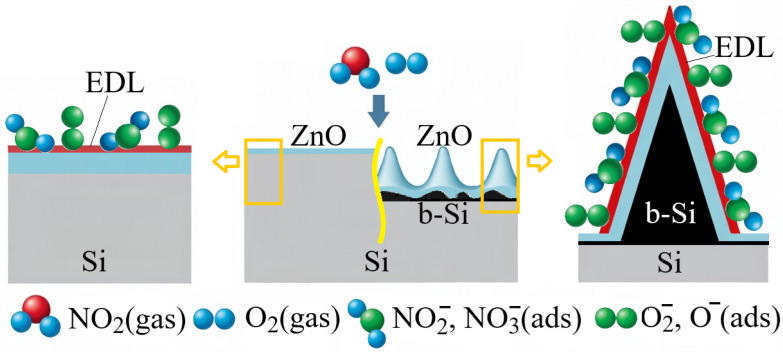
Schematic of NO_2_ adsorption and electron depletion in ZnO/Si (**left**) and ZnO/b-Si/Si (**right**) structures.

Figure 11 schematically illustrates NO_2_ interaction with ZnO/Si (left) and ZnO/b-Si/Si (right). As shown in the schematic diagram, the highly developed morphology and large specific surface area of the b-Si layer provide a significantly larger number of active adsorption sites for both oxygen ions and gas molecules. In addition, the nanostructured geometry facilitates efficient diffusion of gas molecules into the structure and promotes their interaction with the ZnO film covering the sidewalls of the b-Si nanoneedles. As a result, ZnO/b-Si/Si sensors exhibit a substantially higher sensor response compared with planar ZnO/Si structures.

It should also be noted that heterojunction effects at the ZnO/Si interface may potentially contribute to the sensing behavior of the fabricated devices, as previously reported for structures based on porous silicon [21,22]. To examine this possibility, control experiments were performed using sensors fabricated on n-type silicon wafers. The sensor responses measured under identical experimental conditions were found to coincide (within an experimental uncertainty of less than 3%) with those obtained for sensors fabricated on p-type wafers. This observation confirms that the sensing signal is primarily governed by the ZnO sensing layer. The silicon substrate, regardless of its conductivity type, mainly acts as a mechanical, thermal, and morphological support without significantly influencing the gas sensing mechanism.

## 4. Conclusions

We have demonstrated that nanostructured black silicon layers fabricated by reactive ion etching serve as an effective platform for NO_2_ detection using ZnO thin films prepared by a sol–gel method. These layers possess a highly developed nanostructured morphology formed by densely packed silicon nanoneedles, resulting in an effective surface area approximately an order of magnitude larger than that of planar silicon substrates. The fabricated ZnO/b-Si/Si sensors exhibit a response of 448% to 25 ppm NO_2_ at 200 °C, which is about 1.5 times higher than that of planar ZnO/Si sensors. Notably, a comparable sensitivity (~300%) is achieved at a reduced operating temperature of 140 °C, demonstrating the potential for energy-efficient gas sensing. Control experiments confirm that the sensing mechanism is governed primarily by the ZnO film, while the b-Si layer mainly acts as a morphological scaffold that enhances the accessible surface area. The compatibility of the fabrication process with standard microelectronic technologies makes the proposed ZnO/b-Si/Si structures promising candidates for integration into silicon-based smart gas sensor systems. These results highlight the potential of nanostructured b-Si platforms to enable next-generation highly sensitive gas sensors with energy-efficient operation.

## Figures and Tables

**Table 1:** Elemental composition of the samples by EDS (wt.% and at.%).

Element/Structure	Si		O		Zn	
	at. %	wt. %	at. %	wt. %	at. %	wt. %
b-Si/Si	96.53	97.99	3.47	2.01	-	-
ZnO/Si	48.65	43.70	32.34	16.55	19.01	39.75
ZnO/b-Si/Si	42.17	36.99	35.71	17.84	22.12	45.17

## Data Availability

The original contributions presented in this study are included in the article. Further inquiries can be directed to the corresponding author(s).
